# Mass screening tools for glucose-6-phosphate dehydrogenase deficiency: validation of the WST8/1 -methoxy-PMS enzymatic assay in a highly malaria-endemic area in Uganda

**DOI:** 10.1186/1475-2875-11-S1-O28

**Published:** 2012-10-15

**Authors:** Mariana De Niz, Alice C Eziefula, Catherine Maiteki-Sebuguzi, Sam Gonahasa, Deborah DiLiberto, Patrick Tumwebaze, Sarah G Staedke, Chris J Drakeley

**Affiliations:** 1 Department of Immunology and Infection, London School of Hygiene and Tropical Medicine, London, UK; 2 Department of Medicine, Makerere University, Kampala, Uganda; 3 Infectious Disease Research Collaboration, Kampala, Uganda

## Background

Glucose-6-phosphate dehydrogenase (G6PD) deficiency is believed to confer protection against malaria and its distribution and prevalence are geographically correlated with malaria endemicity. This enzymopathy has been identified as the cause of haemolysis following administration of the antimalarial drug primaquine. Screening for G6PD deficiency prior to administration of primaquine together with artemisinin combination therapy for treatment or mass-drug administration is being considered for malaria elimination. Current conventional methods for G6PD screening have limitations for field use.

## Methods

The WST8/1 -methoxy PMS method, recently adapted to assay G6PD activity in a 96-well format using dried bloodspots, was validated using a current gold standard enzymatic assay (R&D Diagnostics Ltd^®^). A study was conducted to identify prevalence of G6PD deficiency in Tororo, a highly malaria-endemic region in Uganda. The performance of the test under various temperature, light, and storage conditions was evaluated.

## Results

The WST8/1-methoxy PMS assay was found to have 72% sensitivity and 98% specificity when compared to the commercial enzymatic assay. Its calculated AUC was 0.904 suggesting good agreement. Most of the cases misclassified had borderline values of G6PD activity either between mild and normal activity values, or between moderate and severe deficiency values. Other misclassifications were related to outlier haemoglobin values. Although severe G6PD deficiency was not found in the area, the test enabled identification of low G6PD activity. The assay was found to be highly robust in terms of light sensitivity, performance under temperature variations, and storage conditions for bloodspots, assay mixes and tested samples.

## Conclusions

The assay was comparable to the currently used standard enzymatic test, yet offered advantages in terms of cost, storage, portability, and use in resource-limited settings. As with other G6PD tests, outlier haemoglobin levels (eg. as a result of recent haemolytic crises) may confound G6PD level estimation.

